# Protective Effects of Restricted Diet and Antioxidants on Testis Tissue in Rats Fed with High-Fat Diet

**DOI:** 10.6091/ibj.1398.2015

**Published:** 2015-04

**Authors:** Amaneh Mohammadi Roushandeh, Iraj Salehi, Motahareh Mortazavi

**Affiliations:** 1 *Research Center for Molecular Medicine, Medicine Faculty, Hamadan University of medical sciences, Hamadan, Iran;*; 2 *Neurophysiology Research Center, Hamadan University of medical sciences, Hamadan, Iran; *; 3 *Dept. of Anatomical Sciences, Medicine Faculty, Hamadan University of Medical Sciences, Hamadan, Iran*

**Keywords:** Spermatogenesis, high-fat Diet, Restricted fat diet, Antioxidants, Astaxanthin

## Abstract

**Background:**

A high-fat diet (HFD) promotes the oxidative stress formation, which in turn has hazardous effects on reproductive system and fertility. The present study examines the potential positive effects of a restricted high-fat diet (RHFD) and antioxidants consumption on sperm parameters and testis tissue in rats.

**Methods:**

Male rats (n = 48) were divided into four groups (12 in each group): control group (Cont), HFD group, RHFD, and RHFD with astaxanthin and vitamins E and C group (RHFDA). After 12 weeks, serum analysis and sperm parameters study were performed. Sections of fixed testes were stained with Hematoxilin and Eosin to study the histological changes. A one-way ANOVA was used to compare the data.

**Results:**

HFD fed animals presented significant increase in weight load and serum low density lipoprotein (LDL-C) levels (*P* < 0.05). The sperm count in RHFD was lower than three other groups (*P* < 0.05) and sperm motility of RHFDA group was significantly higher than HFD and RHFD groups (*P* < 0.05). The histological study was showed a significant increase in spermatogonium number in RHFDA compared to three other groups (*P* < 0.05). The number of spermatocyte I and spermatid in RHFD was significantly (*P* < 0.05) lower than Cont and HFD groups.

**Conclusion:**

HFD and obesity can affect sperm parameters and spermatogenesis and antioxidants consumption may improve their quality. Although the RHFD is a benefit way in weight loss and decrease of LDL-C of serum, but it is suggested that is not effective on sperm quality improvement.

## INTRODUCTION

Obesity, as a consequence of high-fat diet (HFD), is a multifactorial disease. It is characterized by an increased mass of adipose tissue, an active endocrine and secretary organ, and results in diabetes, hypertension, [1], and coronary heart diseases [2, 3]. Furthermore, obesity has hazardous effects on reproductive system and fertility and exerts fertility via the decline of successful pregnancy rates in both natural and assisted conception cycles [4, 5]. It also affects male infertility through decreasing the sperm quantity and motility, thus reducing fertilization rate  [6, 7]. A number of studies have suggested that increase in sperm DNA damage and sperm intracellular reactive oxygen species, which induces oxidative stress, is associated with diet-induced obese male  [7, 8]. A high body mass index and fat accumulation promote the oxidative stress. Although the reactive oxygen species are required for normal sperm function such as capacitation and acrosomal reaction, the excessive levels of reactive oxygen species can negatively impact sperm quality [8].

Therefore, finding strategies to overcome the undesirable consequences of obesity and HFD on male reproductive system is necessary. Restricted high-fat diet (RHFD) and antioxidant consumption may be appropriate ways to decline the negative effects of obesity in obese patients and also to prevent obesity-induced complications without changes in dietary pattern. Caloric restriction is one of the most efficient ways to promote weight loss and is known to activate protective metabolic pathways [9]. A calorie restriction diet, which is a reduction in calorie intake without malnutrition, improves many parameters involved in immune responses and antioxidant enzyme activities. It has been shown that RHFD reduces metabolic abnormalities, such as dyslipidemia and plasma adipokine levels, in the obese rats continuously fed with HFD [1]. In addition, high-fat calorie restriction as well as calorie restriction maintains the redox-balancing power in cells and tissues and exhibits reduced oxidative damage [10].

Antioxidants act as free radical scavengers to protect spermatozoa against reactive oxygen species, such as superoxide dismutase (SOD), catalase, and glutathione peroxidase (GPX). In addition, semen contains a variety of non-enzymatic antioxidant molecules, such as vitamin C, vitamin E, pyruvate, glutathione, and carnitine [11]. These antioxidants compensate for the loss of sperm cytoplasmic enzymes as the cytoplasm is extruded during spermiogenesis, which in turn diminishes endogenous repair mechanisms and enzym-atic defenses [4].

Vitamins E and C play critical roles as non-enzymatic antioxidants. [11]. Vitamin E plays a vital role in protecting cell membranes from trapping oxidative damage as well as scavenging free radicals within cellular membranes. Vitamin C is a water-soluble antioxidant that reduces radicals from a variety of sources and also serves to recycle oxidized vitamin E. 

Astaxanthin  is a ketocarotenoid and belongs to a larger class of phytochemicals known as terpenes, which are built from five-carbon precursors isopen-tenyl diphosphate and dimethylallyl diphosphate. It is classified as xanthophyll, originally derived from a word meaning "yellow leaves". Astaxanthin, a red carotenoid pigment, is a biological antioxidant that is found naturally in a wide variety of living organisms. It has many potent pharmacological effects, including anti-tumoral, anti-diabeti, and anti-inflammatory activities [12-14]. Ikeuchi *et al.* (12) have indicated that astaxanthin may reduce liver weight, liver triglyceride, plasma triglyceride, and total cholesterol and results in reducing body weight and adipose tissue mass due to HFD. However, the chronic effects of astaxanthin as an anti-obesity agent have not been demonstrated. 

The present study was performed to evaluate the possible effect of antioxidants (vitamin E, C, and astaxanthin) on spermatogenesis and testis tissue in rats fed with HFD and RHFD. We hypothesize that the antioxidants and RHFD could improve the sperm quality and testis tissue morphology in rats with HFD.

## MATERIALS AND METHODS

***Animals. ***Albino male Wistar rats (n = 48, three months of age, 200 ± 20 g) were supplied by the Animal Room of Hamadan Uuniversity of Medical Sciences (Hamadan, Iran) and maintained under controlled temperature (± 23°C) and lighting conditions (12 h light:12h dark photoperiod). Animal experiments were approved by the Ethical Committee of Hamadan University of Medical Sciences and performed in accordance with the guideline. Rats were divided into four groups (n = 12 in each group). Control group received normal diet (containing 304 kcal of energy per 100 g) and HFD group received 444 kcal of energy per 100 g. The received colorie of RHFD was 30% of colorie received by HFD group, and the last group received RHFD with astaxanthin and vitamins E and C (RHFDA) (0.2% each of vitamin E and C and 0.6% astaxanthin 10%) for 12 weeks [13]. The HFD was composed of milk fat with approximately 60% fat. Intake of energy, fatty acid, carbohydrate, and protein in animals was calculated during the study. Animals' weight was also measured two days weekly during the study.

***Serum index analysis. ***Blood samples were collected from inferior vena cava and centrifuged at 300 g for 15 min. The level of total cholesterol and serum triglycerides were measured by using standard enzymatic methods. The levels of high density lipoprotein (HDL-C) HDL-C and low density lipoprotein (LDL-C) were determined according to the heparin-nickel and heparin-citric acid kit manufacturer's instructions (Pars Azmoon Co., Tehran, Iran). The level of total serum antioxidants was measured by a commercial kit (Pars Azmoon Co., Tehran, Iran) based on the kit guidelines.

**Fig. 1:**
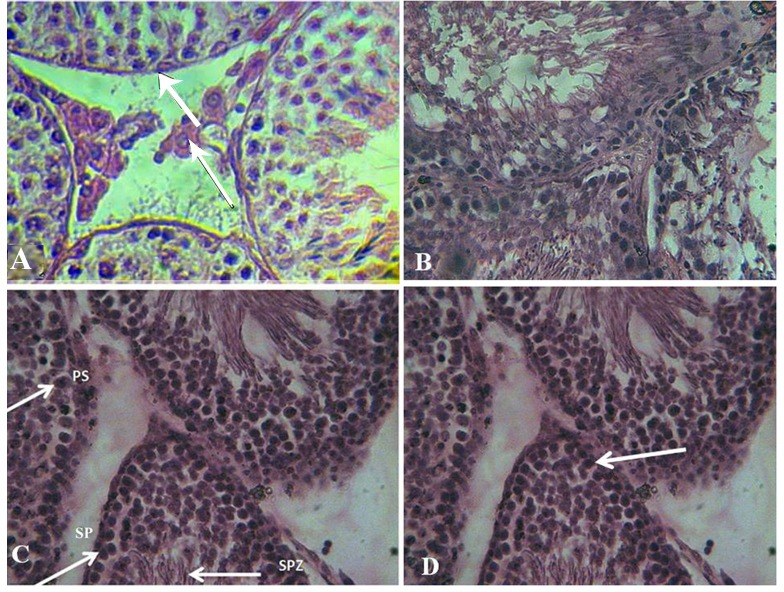
Germinal epithelium of seminiferous tubules in different groups. (A) The tubules in control group. Thick arrow indicates the basement membrane and the thin arrow points to leydig cell in interstitial space. The germ cells arranged normally in basement membrane. (B) Seminiferous tubules in HFD group. There are some vacuoles within the cells. (C) The seminiferous tubules in HFDRA group. PS, primary spermatocye; SP, spermatogonia; SPZ, spermatozoa. (D) The tubules in HFDRA group in which spermatogonia was increased (arrows)

***Semen analysis and histological study. ***Animals were anesthetized and sacrificed after 12 weeks. Sperm was obtained from the tail of epididymis and transferred to Ham's F-10 medium for analyzing viability, motility, and morphology of the sperms with routine protocols. Briefly, after dilution of semen, the number of sperms was counted using a Neubauer chamber. Viability of sperm was determined with eosin staining. Also, motility of sperm was determined in four grades, including hyperactive progressive, progressive, vibrating movement, and immotile. Sperms with normal (intact and typical rat sperms) and abnormal morphology (without head or tail, coiled tail, and pinhead) were calculated to survey sperm morphology. Testes were fixed in 10% formalin, cut into 5-µm sections and stained with hematoxylin and eosin. Seminiferous tubules epithelium (spermato-gonium, primary spermatocyte, spermatid, and sertoli cells) and leydig cells were studied with a light microscope (Fig. 1).

***Statistical analysis. ***A one-way ANOVA was used to compare data, such as weight, sperm parameters, histological data, HDL-C, LDL-C, total cholesterol, and triglycerides among four groups. Post hoc comparisons were performed by Tukey's test. The results were presented as mean ± standard deviation, and *P* value of 0.05 or less (*P* ≤ 0.05) was considered as significant. 

## RESULTS

***Animal weight. ***Animals were weighted during the experiment two days weekly, and weight difference at the beginning and the end of the study was defined as weight load. As expected, the result showed that the weight load of RHFD and RHFDA groups were significantly lower than Cont and HFD groups. Moreover, weight load of HFD group was higher compared to Cont group (*P* < 0.05) (Table 1). 

***Serum analysis. ***The levels of total cholesterol and serum triglycerides of the HFD group were significantly higher than other groups (*P* < 0.05). Also, LDL-C level was higher in RHFD compared to RHFDA groups significantly (*P* < 0.05). The HDL-C/LDL-C level in the Cont group was significantly lower than the three other groups (*P* < 0.05). Furthermore, the level of serum antioxidant was increased significantly in RHFDA group in comparison to Cont, HFD, and RHFD groups (*P* < 0.05) (Table 1). 

***Semen analysis. ***Sperm number in animals fed with RHFD was decreased significantly compared to Cont group (*P* < 0.05). Also, viability and normal morphology of the sperms in the HFD, RHFD, and RHFDA groups were significantly lower than Cont group (*P* < 0.05). The sperm motility of HFD and RHFD groups was decreased, while that of RHFDA group was decreased significantly compared to Cont group but was significantly higher than HFD and RHFD groups (*P* < 0.05) (Table 1). 

***Histological study. ***The number of spermatogonium, primary spermatocyte, spermatid and sertoli cells was studied in germinal epithelium of seminiferous tubules. The number of leydig cells was evaluated adjacent to the seminiferous tubules. As shown in Table 2, the number of spermatogonium of RHFDA group was increased significantly compared to the three other groups (*P* < 0.05). Moreover, the number of spermatocyte I and spermatid in RHFD group was significantly lower than in control and HFD groups (*P* < 0.05). In addition, the number of spermatocyte I in RHFDA was increased significantly compared to RHFD group (*P* < 0.05). Furthermore, the leydig cells in HFD, RHFD, and RHFDA groups were significantly lower than control group (*P* < 0.05). However, no significant differences were observed in the number of sertoli cells among different groups (*P* > 0.05) (Fig. 1 and Table 2).

**Table1:** The weight load, sperm parameters, and serum index in Cont, HFD, RHFD, and RHFDA groups

	**Parameters**	**Cont**	**HFD**	**RHFD**	**RHFDA**
	Weight load (g)	161.9 ± 16.9	192.4 ± 21.2^b^	98.6 ± 24.7^c^	88.9 ± 20.8^c^
**Sperm parameters**	Count (per 1 ml)	53.75 × 106 ± 4.7 × 106	56.35 × 106 ± 6.4 × 106^a^	39.5 × 106 ± 5.6 × 106^b^	51.1 × 106 ± 7.2 × 106 a
	Viability (%)	98.9 ± 0.97	97.3 ± 1.5^b^	97.0 ± 1.6^b^	96.5 ± 1.8^b^
	Motility (%)	65.1 ± 5.5	56.3 ± 6.07^b^	52.5 ± 6.2^b^	58.7 ± 8.4^c^
	Normal morphology (%)	98.7 ± 0.56	96.1 ± 1.2^b^	96.8 ± 1.2^b^	96.7 ± 1.6^ b^
					
**Serum ** **indices**	HDL-C	16.62 ± 2.6	25 ± 3.5^b^	28.5 ± 5.6^b^	22.42 ± 3.4 b
	LDL-C	39.7 ± 5.4	41.4 ± 7.5^b^	44.6 ± 6.3^b^	38.75 ± 8.1^a^
	HDL-C/LDL-C	0.43 ± 0.1	0.62 ± 0.14^b^	0.64 ± 0.1^b^	0.60 ± 0.11^b^
	Total cholesterol	66.25 ± 4.9	108.75 ± 18.5^b^	78.75 ± 5.1^a^	69.3 ± 6.8^a^
	Triglyceride	60.25 ± 5.5	167 ± 12^b^	89.1 ± 17.24^a^	63.1 ± 8.8^a^
	Level of serum antioxidants	0.15 ± 0.02	0.18 ± 0.02^a^	0.15 ± 0.02 a	0.22 ± 0.02^b^

a showes the insignificant difference with control group (*P > 0.05*),

b showes the significant difference with control group (*P < 0.05*,

c showes the significant difference with control and the other experimental groups (*P < 0.05*).

## DISCUSSION

Several studies have reported that men with a HFD or obese men have lower quality and quantity sperm, which result in low fertility rate, in comparison with healthy men [15]. As early as the 10^th^ century, Avicenna, a Persian scientist, described the negative effects of obesity on male fertility in his encyclopedic medical book, the Canon of Medicine [5, 15-17]. Therefore, the present study was performed to evaluate the possible effects of RHFD and antioxidant consumption on seminiferous tubule epithelium, sperm quality, and serum indices in the rats fed with a HFD. We hypothesize that caloric restriction and antioxidant consumption could improve the sperm quality. Furthermore, some studies have indicated and association between the body mass index and reproductive parameters in men [5, 15, 18]. According to our data, the body weight load in RHFD and RHFDA groups was lower than the two other groups, whie it was higher in HFD group in comparsion with other groups. These results demonstrate the positive effect of calorie restriction on prevention of body weight raise and support the results obtained by Park *et al.* [10], who evaluated the possible effect of HFD calorie on the induction of inflammation and oxidative stress damage. As expected, HFD resulted in the elevation of the level of serum lipids, such as cholesterol, triglyceride, and LDL-C. The serum analysis showed that the restriction of HFD could lead to a significant reduction in the levels of total cholesterol and serum triglyceride. In addition, after antioxidant consumption, the level of LDL-C was decreased. Our data suggests that caloric restriction could decrease the serum levels of total cholesterol, triglyceride, and LDL-C. 

We found that HFD and RHFD decrease the motility, viability, and normal morphology of sperm; however, the antioxidant consumption caused to significant increase in sperm motility in relation to HFD and RHFD groups. Accumulation of a large amount of fat in the scrotum of obese subjects could cause overproduction of oxidative stress. Therefore, excessive production of reactive oxygen species in turn results in the damage of spermatogenesis [19]. These results are in line with the findings on protective effects of antioxidants on sperm quality against oxidative stress [20, 21]. It seems that the present designed RHFD in our study is not enough to protect the unfavorable effects of HFD on sperm quality, and more studies are necessary to obtain an optimal diet in this field. MacKay *et al.* [22] demonstrated deleterious effect of caloric restriction on testicular gene expression, which in turn could impact on sperm maturation and function. 

Moreover, our histological study has revealed that the number of spermatogonium as well as spermatocyt I in RHFD group is increased via antioxidant consumption, but HFD and RHFD has no negative effects on spermatogonium and spermatocyt I. As Vigueras-Villaseñor *et al.* [19] reported, there were not any structural alterations in the seminiferous tubules in obese rats. One possible explanation would be the normal sertoli cell activity for the normal process of spermatogenesis [19]. In the current study, no significant difference was observed between the number of sertoli cells among control and experimental groups; hence, spermatogenesis was not affected. Additionally, according to an interesting observation by Ghanayem and co-workers [15], spermatogenesis is affected only in males with extreme obesity. However, more studies required to reveal HFD and its effects on different systems, especially reproductive one.

**Table 2:** The number of germinal epithelial cells of semi-niferous tubules (spermatogonia, primary spermatocyte, spermatid, sertoli and leydig cells) in cont, HFD, RHFD, RHFDA groups

**Germinal epithelial cells**	**Cont**	**HFD**	**RHFD**	**RHFDA**
Spermatogonium	13.42	14.6^a^	14.8^a^	15.42^b^
Spermatocyte I	24.6	25.8^a^	20.5^b^	24^a^
Spermatid	22.8	20^a^	14.6^b^	17.75^b^
Sertoli cell	5	4.42^a^	5.1^a^	4.3^a^
Leydig cell	11.2	9^b^	9.6 b	10.8^b^

a shows the insignificant difference with control group (*P > 0.05*),

b showes the significant difference with control group (*P < 0.05*)

In another view, it has been suggested that the phenomenon of increased apoptosis mediated by oxidative stress is observed in leydig cells showing a decline in HFD [11]. In the same manner, as shown in the present study, HFD and obesity and subsequently oxidative stress induction could alter directly the spermatozoids production via damage to motility, normal morphology, and viability [23]. Thus, any excess reactive oxygen species must be inactivated continuously in order to have normal sperm function. This function is performed by the antioxidants of the seminal plasma. The impaired antioxidant defense mechanisms and/or excessive production of reactive oxygen species lead(s) to oxidative stress [11]. In the present study, we have demonstrated a significant reduction in sperm quality in association with HFD. It has been displayed that antioxidants, such as vitamins E and C and astaxanthin might help to preserve the functional competence of spermatozoa subjected to an oxidative attack. The protection of sperm DNA against oxidative damage via antioxidants has been supported by several *in vitro* studies, and that the percentage of motile sperm in a semen sample is correlated with sperm antioxidants content  [4, 24]. Also, Shalaby *et al.* [25] have proved the protective effect of antioxidants against sperm damage in an animal study of male rats fed with HFD containing 1% cholesterol. The fertility of rats was reduced due to the diet-induced hyper-cholesterolemia and the concomitant administr-ation of a-tocopherol (vitamin E) and the hypolipidemic and hypocholesterolemic drug simvast-atin improved the rat's fertility. The antioxidant treatment acts as a spermatozoa preservative against lipid peroxidation, restored membrane fluidity and so sperm function and fertilizing capacity. Furthermore, the concept that antioxidant therapy may be profitable for sperm quality is supported by a systematic review [21].

The results of the current investigation support the hypothesis that HFD and obesity are risk factors in male infertility. Although the process of spermatogenesis was not altered by HFD in our study, the sperm quality was reduced. On the other hand, RHFD is an effective way to decrease body weight and serum lipids; neverthless, it seems that it is harmful to sperm function as viability, normal motility, and morphology. 

In conclusion, based on our study, HFD leads to deficiency in sperm function and male fertility, and antioxidant therapy may be a profitable way for improvement of sperm quality. However, the effects of restricted diet on male germ cells are in doubt and need more investigation.
